# Focus on Adoptive T Cell Transfer Trials in Melanoma

**DOI:** 10.1155/2010/260267

**Published:** 2010-12-26

**Authors:** Liat Hershkovitz, Jacob Schachter, Avraham J. Treves, Michal J. Besser

**Affiliations:** ^1^Ella Institute of Melanoma, Sheba Medical Center, Tel-Hashomer 52621, Israel; ^2^Sheba Cancer Research Center, Sheba Medical Center, Tel-Hashomer 52621, Israel; ^3^Department of Human Microbiology and Immunology, Sackler School of Medicine, Tel Aviv University, Tel Aviv 69978, Israel

## Abstract

Adoptive Cell Transfer (ACT) of Tumor-Infiltrating Lymphocytes (TIL) in combination with lymphodepletion has proven to be an effective treatment for metastatic melanoma patients, with an objective response rate in 50%–70% of the patients. It is based on the *ex vivo* expansion and activation of tumor-specific T lymphocytes extracted from the tumor and their administration back to the patient. Various TIL-ACT trials, which differ in their TIL generation procedures and patient preconditioning, have been reported. In the latest clinical studies, genetically engineered peripheral T cells were utilized instead of TIL. Further improvement of adoptive T cell transfer depends on new investigations which seek higher TIL quality, increased durable response rates, and aim to treat more patients. Simplifying this therapy may encourage cancer centers worldwide to adopt this promising technology. This paper focuses on the latest progress regarding adoptive T cell transfer, comparing the currently available protocols and discussing their advantages, disadvantages, and implication in the future.

## 1. Introduction

Metastatic melanoma is a highly aggressive cancer. It is the sixth most common cancer type in men and the seventh in woman and has a poor prognosis with a median survival of 6–10 months and a 5 year survival rate of about 10% [1, 2]. So far, only two drugs have been approved by the Food and Drug Administration (FDA) for the treatment of metastatic melanoma patients, the chemotherapeutic agent dacarbazine (DTIC) and the immunomodulator Interleukin-2 (IL-2). DTIC is an antineoplastic chemotherapy drug used in the treatment of various cancers [3, 4], which alkylates and crosslinks DNA during all phases of the cell cycle, resulting in disruption of DNA function, cell-cycle arrest, and apoptosis [5]. DTIC as a single agent can induce objective tumor regression in 15%–20% of metastatic melanoma patients with median response duration of 5 to 6 months and a negligible complete response rate [6]. The administration of IL-2 activates endogenous antitumor reactive T cells and NK cells resulting in a 20%–30% objective response rate, with complete regression in only 5%–8% of the melanoma patients [7–9]. Chemobiotherapy, combining low-dose IL-2 and chemotherapy, demonstrates a similar response rate to that of high-dose IL-2 alone, with 7% of the patients achieving a durable complete response [10, 11].

In the past decade, various therapeutic approaches for metastatic melanoma patients have been tested, most of them with unsatisfactory clinical results. The discovery of melanoma-specific tumor associated antigens paved the way for the use of immunotherapy [12]. Immunotherapy is an innovative approach which includes: (1) cytokines, such as IL-2 and IFN*α*, which stimulate immune effector cells, (2) monoclonal antibodies, against cytotoxic T lymphocyte-associated antigen 4 (CTLA-4) or programmed death 1 (PD-1), (3) intratumoral gene transfer with genes encoding for example for granulocyte-macrophage colony-stimulating factor (GM-CSF), (4) active immunization via vaccination with peptides, dendritic cells, recombinant viruses encoding tumor-associated antigens (TAA), and costimulatory molecules or whole tumor cell vaccines, and (5) adoptive T cell transfer.

Like IL-2, IFN*α* is FDA approved, but only for adjuvant treatment of stage III melanoma patients and not for patients with metastatic disease [11]. The monoclonal antibody ipilimumab, which blocks the inhibitory effects of CTLA-4 on T cells, was evaluated in a randomized phase III study and showed an improved overall survival compared to glycoprotein 100- (gp100-) derived peptide vaccines. However, adverse events were sometimes severe or long lasting [13].

In the past few years, the field of intratumoral gene transfer has been established. A very encouraging phase II study conducted on 43 melanoma patients reported an almost 30% objective response with very minor side effects following intratumoral injection of a viral vector encoding for GM-CSF [14]. On the other hand, various cancer vaccines trials have failed so far, as they demonstrated a very low objective response rate of only 3%-4% [15].

Among the current immunotherapy approaches, ACT therapy using autologous tumor infiltrating lymphocytes (TIL) has shown to be an effective treatment for patients with metastatic melanoma and can mediate objective response rates between 50%–70% with manageable toxicity [16–18]. Furthermore, the ability to genetically engineer tumor antigen specific lymphocytes may enable the use of ACT to a wider patient population and for treatment of other malignancies. This paper summarizes and compares the different ACT approaches and discusses their advantages, disadvantages, and challenges.

## 2. Initial ACT Studies

Adoptive cell transfer immunotherapy relies on the transfer of *ex vivo* expanded and activated tumor-specific lymphocytes to the autologous tumor-bearing host. In the 1980s, the availability of recombinant IL-2 for *in vitro* use brought the breakthrough of ACT trials [19–21]. Using IL-2 as a T cell growth factor in culture promoted the ability to expand human lymphocytes to larger scales and for longer periods. The first generation of ACT utilized lymphokine-activated killer (LAK) cells with high-dose IL-2 for the treatment of patients with advanced cancer but was not shown to be superior to the treatment of IL-2 alone [22]. *In vitro* lysis or cytokine release assays revealed that expanded LAK cells did not exhibit any anti-tumor activity [23].

The second generation of ACT employed tumor-infiltrating lymphocytes instead of LAK cells. TIL are T cells present within the tumor of the patient, which have the ability to specifically recognize tumor antigens and thereby eliminate malignant cells. However, in most cancer patients, those naturally occurring TIL fail to destroy the tumor as they are outnumbered, not fully activated, or suppressed. Therefore, the main objective of TIL-ACT is the production of large numbers of activated tumor-specific T cells. Furthermore, patients receive IL-2 immediately after TIL infusion to further activate the administered TIL. In most current protocols, high-dose intravenous bolus IL-2 (720,0001 IU/kg) is administered intravenously every 8 hours to tolerance or to a maximum of 15 doses.

An initial TIL-ACT trial for metastatic melanoma patients was published in 1988 and summarized in 1994 by Rosenberg et al. at the National Cancer Institute (NCI), Bethesda [24, 25] (Table 1, row A). In this study, 86 consecutive patients were treated with autologous TIL including high-dose intravenous bolus IL-2. TIL single-cell suspension was generated by the enzymatic digestion of the entire tumor and expanded in a high concentration of IL-2. After 2-3 weeks, the cultures were cleared of tumor cells and lymphocytes were further expanded until they reached a number of 10^9^ cells. Then, the cells were transferred from tissue culture plates to cell culture bags and cultured until at least 10^11^ TIL were generated. TIL and IL-2 were administered in two cycles separated by approximately 2 weeks. Fifty-seven of the 86 patients received an additional single low dose of cyclophosphamide (25 mg/kg) approximately 36 hours before receiving the first TIL infusion. The overall objective response rate was 34% and comparable to studies with high-dose IL-2 alone (31%) or IL-2-based chemobiotherapy (35%). Only 5 of the patients experienced a complete response, and the median survival of all partial responders was just 4 months. Analysis of the survival of the transferred cells *in vivo* using cells genetically labeled with neomycin phosphotransferase demonstrated a lack of persistence of the infused cells. The persistence of the TIL in the circulation was barely 0.1% one week after administration [26]. Nevertheless, this initial study demonstrated that TIL-ACT can indeed cause clinical remission although response rates were low at that time.

A retrospective analysis comparing TIL characteristics of responding versus nonresponding patients revealed that responders were treated with TIL that spent less time in culture, had shorter doubling times, and exhibited higher *in vitro* lysis against autologous tumor targets [27, 28]. Additionally, patients who received TIL generated from subcutaneous tumor lesions had higher response rates (49%) compared to those receiving TIL from lymph nodes (17%), probably as lymph nodes contain many unspecific T cells [27].

The results of this study indicated the importance of a suitable *in vitro* selection process which can detect anti-tumor specific TIL.

This pioneer trial paved the way for new clinical studies designed to improve the TIL anti-tumor specificity, the growth and expansion conditions, and the creation of younger TIL culture with higher persistence and lower senescence.

## 3. Current TIL Studies

### 3.1. The “Selected-TIL” Protocol

Earlier studies utilized bulk TIL cultures that contained heterogeneous populations of lymphocytes, only some of which showed anti-tumor activity *in vitro* [24, 25]. Studies of the T-cell receptor rearrangements in TIL revealed the variability of T cells present in these cultures and emphasized the need to develop culture techniques that specifically select anti-tumor reactive cell cultures without growth of other bystander cells that do not contribute to the therapeutic effect [29]. It is known that T cells recognize tumor antigens in an MHC-restricted manner when the appropriate human leukocyte antigen (HLA) restriction element is present [30–32]. Upon antigen recognition cytotoxic T cells secrete the cytokine IFN*γ* which can be measured by a simple enzyme-linked immunosorbent assay (ELISA). The integration of an IFN*γ* release-based *in vitro* assay, which allows the selection of antigen-specific TIL is the core element of the Selected-TIL protocol (Table 1, row B) [33–35].

The generation of Selected-TIL with specific reactivity against tumor antigens consists of three stages.

(1) *Isolation and generation of TIL from the tumor*.

Several methods can be used for initiating TIL and melanoma cultures from the resected tumor. TIL cultures are usually established by cutting the resected tumor specimens into 1–3 mm^3^ fragments from different areas of the tumor. Approximately 8–10 single fragments are placed into different wells of a tissue culture plate, and each obtained TIL culture is maintained independently. This method generates multiple TIL cultures and enables the *in vitro* screening of every individual culture. Besser et al. showed that for 97% of the patients at least one TIL culture can be generated whereas a primary melanoma cell culture can be established from 70% of the resected tumor [36]. Generating TIL cultures from a single small fragment and expanding those cells to approximately 50 × 10^6^ cells, a suitable number to initiate large scale expansion, requires 21–36 days.

(2) *In vitro TIL selection*.

Each individual TIL culture is screened for tumor specific reactivity by coincubation of TIL with autologous melanoma cells over night, followed by the measuring of IFN*γ* levels. Alternatively, a HLA-A2 matched melanoma cell line can be used for TIL cultures from HLA-A2 positive patients. TIL pass the screening process, if IFN*γ* secretion exceeds 200 pg/ml and double the level of a control in which the TIL are coincubated with any HLA-mismatched melanoma cell line [37, 38]. Only individual TIL cultures complying with this criterion can be used for further large-scale expansion.

(3) *Large-scale expansion*.

After reaching 50 × 10^6^ cells, TIL are massively expanded, resulting in an approximately 1000-fold expansion and a final cell number of about 50 × 10^9^ TIL. This rapid expansion process (REP) requires anti-CD3 antibody, irradiated peripheral blood mononuclear feeder cells and IL-2. The large-scale expansion process is a standardized procedure which requires 14 days [36, 37] and results in the production of high numbers of activated TIL ready for administration.

Response rates are assessed using the Response Evaluation Criteria In Solid Tumors (RECIST) guidelines [39] and Objective Responses (OR) include Complete Remissions (CR) and Partial Responses (PR).

A major breakthrough in the field of ACT was the addition of lymphodepleting NonMyeloablative Chemotherapy (NMC). NMC is given in order to suppress endogenous regulatory T cells and to provide the optimal environment for the infused TIL. Various mouse models have shown the marked effect of lymphodepletion on the efficacy of T cell transfer trials (reviewed in [40]). In 2002, Dudley et al. conducted a Selected-TIL study in which all TIL patients received NMC starting 7 days prior TIL infusion (day 0). On days −7 and −6, patients were treated with cyclophosphamide (60 mg/kg) and on days −5 to −1 with fludarabine (25 mg/m^2^). Six of 13 (47%) patients achieved an objective response with significant shrinkage of the tumors (Table 1, row B) [41]. The addition of NMC before TIL administration resulted in the persistent repopulation of anti-tumor T cells in the patients, with proliferation of functionally active transferred cells *in vivo* and the trafficking to tumor sites. A further study was conducted by Dudley et al. three years later including 35 patients, reported 18 (51%) patients that experienced OR including three ongoing complete remissions and 15 (42%) partial responses with a mean progression-free survival of 11.5 months [17]. The toxicities associated with this treatment included the expected toxicities of high-dose IL-2 therapy, such as hypotension, pulmonary congestion, vascular leak syndrome, and bone marrow suppression associated with lymphodepleting chemotherapy. Some patients exhibited autoimmune manifestations, such as vitiligo and uveitis, caused by destruction of melanocytes in skin and eyes. Toxicities were mostly transient and readily managed by standard supportive techniques with no treatment-related deaths.

The Selected-TIL protocol including lymphodepleting preconditioning of the patient was indeed a major improvement to earlier TIL protocols. Though, the main disadvantage of this approach was the extremely high dropout rate of enrolled patients. In a report conducted in 2009, it was shown that about two thirds of all enrolled patients had to be excluded from the treatment [36]. This high dropout rate is the result of two major reasons, directly related to the *in vitro* IFN*γ* screening process: (1) failure to establish an autologous melanoma line for HLA-A2 negative patients which is essential to perform the screening (approximately 30%) and (2) absence of TIL cultures secreting IFN*γ* (approximately 40%) [16, 36]. A few more patients were excluded due to clinical deterioration or as TIL cultures could not be established [36].

Due to the high dropout rate, a different criterion which relates to clinical response was required. Interestingly, in 1994, Schwartzentruber et al. could not correlate IFN*γ* secretion to clinical outcome [27]. On the other hand, characteristics that were repeatedly reported to have a significant positive association to clinical response were short TIL culture duration and telomere length [27, 28, 42–44]. An *in vitro* study comparing younger TIL cultures to older IFN*γ*-Selected-TIL showed that TIL spending less time in culture have longer telomeres and high levels of the costimulatory molecules CD27 and CD28, which can lead to persistence *in vivo* as well as objective responses [42–46]. These *in vitro* data led to the development of the so called Young-TIL protocol [16, 36].

### 3.2. The “Young-TIL” Protocol

The Young-TIL protocol utilizes bulk unselected TIL, which spend minimal time in culture (Table 1, row C). In contrast to the Selected-TIL protocol, Young-TIL are initiated from a single cell suspension obtained after enzymatic digestion of the resected tumor and not from multiple small fragments. Consequently, the number of TIL is higher in the initial culture which enables the generation of a TIL culture ready for expansion in only 10 to 18 days [45]. Young-TIL are considered ready for expansion when they reach a number of at least 50 × 10^6^ cells and all melanoma cells are eliminated. The process of growing a single TIL culture enormously simplifies the laboratory procedure and lowers costs. In a study from 2010, Besser et al. showed that Young-TIL cultures were successfully established for nearly 90% of the patients (Table 1, row C). As no further selection process was required, all established Young-TIL cultures were eligible for treatment. This resulted in a dramatic improvement of the proportion of treated patients compared to the Selected-TIL protocol with a drop-out rate of only 26%, mainly caused by clinical deterioration of patients during TIL preparation [16].

TIL large-scale expansion, preconditioning of the patients with NMC and administration of high-dose IL-2 following cell transfer were identical to Selected-TIL ACT. IL-2 and chemotherapy-related toxicities as well as response rate were comparable to the Selected-TIL protocol. Ten of 20 (50%) metastatic melanoma patients experienced a clinical objective response including two ongoing CR and eight PR [16]. The significant correlation between short culture duration and clinical response was confirmed. All responders received TIL cultures which were established within 19 days, which then continued to the large-scale expansion process.

In conclusion, Young-TIL ACT as compared to Selected-TIL ACT has the major advantage of being less labor intensive, requires less laboratory expertise, enables the treatment of most enrolled patients, and still results in an objective response rate of 50%.

This process simplification might be essential as it may integrate ACT to more cancer centers worldwide and thus expose new patients to this effective therapy.

### 3.3. “Selected-TIL” ACT with Addition of Total Body Irradiation

Studies on murine ACT models have demonstrated the need for lymphodepletion prior to TIL transfer in order to eliminate suppressive CD4^+^CD25^+^ T-regulatory cells as well as normal endogenous lymphocytes that compete with the transferred cells for homeostatic cytokines such as IL-7 and IL-15. These studies reported a significant correlation between the intensity of lymphodepletion and the *in vivo* antitumor effect of the infused cells [40, 47–50]. Nonmyeloablative chemotherapy using cyclophosphamide and fludarabine are given to deplete the lymphoid compartment of patient and to provide the optimal environment for the infused anti-tumor lymphocytes. Addition of total body irradiation (TBI) can further augment lymphodepletion.

In order to validate if enhanced lymphdepletion can further increase response rate, a series of three nonrandomized clinical trials were conducted with a total of 93 metastatic melanoma patients between the years 2002 and 2008 [17, 18, 41]. In all three studies, TIL were established according to the Selected-TIL protocol. Until 2008 a total of 43 patients were treated with the Selected-TIL protocol in combination with NMC at the NCI, Bethesda, of which the result of the first 35 patients were discussed earlier [17] (see Section 3.1, Table 1, row B). In a second trial, 25 patients received 2-Gy TBI in addition to NMC one day before cell administration (Table 1, row D). In the third study, 25 patients received a total of 12-Gy TBI, 2-Gy was given twice a day for three consecutive days in addition to NMC (Table 1, row E) [18]. All TBI patients received autologous purified CD34^+^ hematopoietic stem cells one or two days after TIL infusion.

Objective responses were demonstrated in 49% (21 of 43) of patients receiving NMC alone, 52% (13 of 25) of the patients treated with NMC and 2-Gy TBI, and 72% (18 of 25) among patients with NMC and 12-Gy TBI [18]. The highest rate of complete remissions was achieved in the 12-Gy TBI group with 28%, as compared to 8% and 6% in the 2-Gy TBI and the no TBI-treated patients, respectively. The 2-year survival rates were 42% and 30% in the trials with 2-Gy TBI and no TBI, respectively, while followup was too short in the 12-Gy TBI group. Patients treated with 12-Gy TBI demonstrated a 1-2 days delay in marrow recovery compared to all other protocol patients and required significantly more blood product support than patients treated with 2-Gy TBI. Furthermore, several toxicities such as fatigue, anorexia, and weight loss were more prolonged in patients treated with 12-Gy TBI, with returning to normal daily routines by 2-3 months, compared to one month for none TBI patients.

It was confirmed that increased lymphodepletion was associated with increased circulating levels of homeostatic cytokines IL-7 and IL-15 with 12-Gy TBI achieving the highest concentrations of the serum cytokines [18]. In addition, it was shown that OR was correlated with telomere length of the transferred cells.

A major disadvantage of the TBI trials was again the high drop-out rate. Alike the Selected-TIL protocol the majority of patients did not have TIL applying to the IFN*γ* criterion. Additionally, transfusion of autologous CD34^+^ stem cells is required after TBI, but sufficient numbers of stem cells could not be isolated for every patient, thus increasing the drop-out rate even more.

Overall, using NMC was proven as a necessary and essential step in the ACT therapy and TBI was shown to augment lymphodepletion and thereby increases the response rate. However, this conclusion must be interpreted with caution as this was not a randomized trial. Statistically, the 72% response rate achieved in 12-Gy TBI group was not significantly different from the 52% or 49% response rate seen in the other groups, but induced severe additional toxicities. Therefore, this study should be continued with more patients to verify the high response rate.

## 4. Genetically Engineered T Cells

One of the possibilities for improving ACT for metastatic melanoma and other cancer patients is based on the transfer of genetically modified peripheral T cells instead of TIL. Genetically engineered T cells may overcome several disadvantages of the TIL protocol. The TIL protocol encounters several difficulties, for example, generating TIL cultures is a technical challenge and requires high laboratory skills [36]. Using this approach, patients have to undergo surgery in order to resect tumor tissue for TIL isolation and TIL are a heterogenic cell mixture with unknown antigen specificity. In addition, the endogenous T cell repertoire is not always responsive to defined tumor-associated antigens such as self-antigen due to developed tolerance [51, 52]. Finally, the drop-out rates of enrolled patients can be quite high, as discussed earlier [36]. These disadvantages led to the investigation of an alternative method producing tumor-reactive T cells. The idea is to create T cells with desired antigen specificity and thereby to enhance the effectiveness of ACT therapy [53, 54]. Genetic modification of T cells is based on the generation of tumor-targeted T cells through the genetic transfer of antigen-specific receptors, which consist of either MHC-restricted T cell receptors (TCR) or non-MHC-restricted synthetic chimeric antigen receptors (CAR) [55]. The genetic approach is more convenient to the patients as it skips surgery and only requires patient's leukopheresis to obtain peripheral T cells for genetic modification.

### 4.1. Artificial *αβ* T Cell Receptors

This approach utilizes the transfer of cloned cDNAs encoding the *α* and *β* chains of a tumor antigen-specific TCR into peripheral T cells by an integrating vector and thus enables the production of large numbers of tumor specific T cells [53, 54]. In an initial clinical trial, metastatic melanoma patients were treated with autologous T lymphocytes transduced with retroviruses encoding TCR with low affinity for melanoma-associated antigen MART-1 [56]. These lymphocytes were administrated to patients preconditioned with NMC followed by high-dose bolus IL-2 (Table 1, row F). Two of 17 patients (12%) underwent regression of liver and lung metastases and exhibited sustained levels of circulating engineered cells one year post-infusion. All the other patients exhibited durable cell engraftment of at least 2 months postinfusion, but did not respond. This study demonstrates the feasibility of using genetically engineered T cells for ACT.

Although response rates were still low (12%) as opposed to 50%–70% in TIL-ACT therapy, this was the first evidence that genetic-engineered T cells can indeed induce clinical response in advanced cancer patients. This encouraging approach is only at its beginning and still needs to be improved. Some of the approaches that may increase the expression and function of the transgene are being studied, including the use of different vectors, the introduction of powerful promoters specific to T cells, and the employment of higher-affinity TCRs. Latest clinical trials are testing new TCRs that recognize a broad array of cancer antigens such as p53, gp100, carcinoembryonic antigen and the NY-ESO-1 antigen of the cancer testis [57–59], which may enable targeting of other cancer types in addition to melanoma.

The main disadvantage of using genes encoding TCR that target just one tumor antigen is that they are likely to be less effective. Tumors are highly heterogeneous and antigen expression differs markedly between tumors not only among individual patients, but even within deposits of the same patient. Investigators should also take into consideration the potency of antigen/MHC downregulation or loss in tumors. Another limitation of this approach is its HLA-restriction.

In 2009, Johnson et al. conducted a clinical study with 36 patients treated with high affinity human TCR to MART-1 (Table 1, row G) and mouse TCR to gp100 (Table 1, row H) [60]. Objective cancer regressions were seen in 30% (6 of 20) and 19% (3 of 16) of patients who received TCR to MART-1 and gp100, respectively. TCR-transduced cells persisted at high levels in the blood of all patients one month after treatment. However, patients exhibited destruction of normal melanocytes in the skin, eyes, and ears and sometimes required local steroid administration in order to treat uveitis and hearing loss [60]. This observation suggests that great caution must be taken in the selection of target antigens to prevent severe toxicity of normal tissues that express the same antigen.

### 4.2. Chimeric Antigen Receptor (CAR)

CARs combine antigen specificity and T cell activating properties in a single fusion molecule. The CAR construct which is retrovirally inserted into normal T lymphocytes, is generated by the fusion of the variable region of the heavy and light chains of a monoclonal antibodies with the T cell signaling chains derived from CD28, 41BB, or CD3*ζ* [61]. While, clinical studies in patients with various cancers treated with CAR have induced only modest responses [62–64], a diverse array of receptors with different functional properties are now rapidly expanding and may provide a more significant assessment for this approach [65–67]. Since CARs bind the tumor antigen in an HLA-unrestricted manner, they are resistant to tumor escape mechanisms related HLA down-regulation and broadly applicable to patients irrespective of their HLA type [63, 64, 68].

To date, there are no published clinical studies using CAR in ACT therapy for melanoma; nevertheless, *in vitro* studies utilizing chimeric receptor against melanoma antigens such as ganglioside GD2, GD3, and MAGE-A1 were reported [69–71]. Lately, a preclinical study using SCID mice showed that the overexpression of GD2 by human melanoma cells allows these cells to be targeted *in vitro* and *in vivo* by GD2 CAR-expressing T cells [69]. Moreover, this study demonstrated that incorporation of endodomains from both CD28 and OX40 molecules mediate costimulation of T lymphocytes, inducing T cell activation, proliferation, and cytotoxicity against GD2-positive melanoma cells.

A major concern of the CAR approach is the toxicity profile. T cells encoding CAR for tumor antigens may be highly potent, but as most tumor-associated antigens are expressed on normal tissues as well, the potential for toxicity is obvious. A clinical study with renal cell carcinoma patients had to be detained as three patients developed cholestasis due to the high expression of the target antigen, carbonic anhydrase on normal tissue [63]. A case report on a metastatic colon cancer patient using CAR targeting the ERBB2 tumor antigen, described respiratory distress of the patient within 15 minutes after cell infusion and displayed a dramatic pulmonary infiltrate on chest X-ray [72].

In summary, T cells transduced with genes encoding for artificial TCR or CAR, enforce tumor antigen recognition, improve T cell survival, generate memory lymphocytes, and reduces T cell death, anergy and immune suppression [55]. Another important advantage of these approaches is the use of patients' circulating lymphocytes with no need of surgical resection and the ability to produce any desired vector. However, this approach demands high laboratory skills and expertise and requires longer culture durations resulting in high costs. The toxicity profile must be improved and more trials need to be conducted to establish this technology.

## 5. Future Challenges of ACT

In order to improve ACT for the treatment of human cancer extensive numbers of studies are being performed. Clinical trials for different ACT studies that are currently recruiting patients are summarized in Table 2. Perhaps the most important trial on this list is a randomized phase II study comparing CD8 enriched Young-TIL ACT to treatment with high-dose IL-2 alone (Table 2, NCT01118091). In fact, this is the first randomized study which will finally reveal the true value of TIL ACT versus standard of care. Many ACT centers worldwide await the results of this study with great interest.

Many studies aim at identifying both patients and cell parameters that are associated with objective clinical responses. In this chapter, we will discuss some of them, including (1) the use of less differentiated T cells, (2) identification of the exact T cell subpopulation responsible for response, (3) combination of ACT with monoclonal antibodies, (4) genetic engineering of new vectors, and (5) reduction of toxicity.

The persistence of the transferred cells has been clearly correlated to clinical response in previous reports [42–46]. One of the factors that influence persistence is the differentiation state of the transferred T cells. Mouse models showed that early effector T cells mediate better anti-tumor response than intermediate and late effector T cells due to their high proliferative and survival potential and their ability to secrete IL-2 [73, 74]. Studies in humans also support this preclinical finding showing that less differentiated T cells are more suitable for ACT [42, 44, 75]. Long telomere length and high CD27 expression are markers of less differentiated cells which appear to correlate with persistence and anti-tumor response [42, 46, 76]. Addition of molecules inhibiting differentiation during T cell expansion, modification of the large-scale expansion protocol, transduction of TCR or CAR into less differentiated cells, or the use of other cytokines besides IL-2 may improve the quality of administrated cells. IL-2 has been shown to be an effective T cell growth factor but also to promote the terminal differentiation of T cells leading to activation-induced cell death [73, 77]. Therefore, the benefit of other cytokines such as IL-7, IL-15, or IL-21 administration alone or in addition to IL-2 should be examined, as recent studies suggest that they may play important roles in manipulating the final T cell phenotype and differentiation status [78–80].

Other efforts are aimed at identifying specific TIL subpopulations responsible for clinical effectiveness. To date, little is known about the underlying composition and cellular interactions which determine the degree of TIL reactivity and consequently how to control TIL reactivity. Oved et al. preformed a study measuring the subpopulation frequencies of TIL populations originating from different melanoma patients [81]. This study showed that the frequency of a single subpopulation cannot predict the collective TIL reactivity to cancer cells* in vitro*. However, by using a simple computational model, one can generate a set of mathematical rules that accurately predict the degree of TIL reactivity in terms of its subpopulation constituents with 91% sensitivity. In addition, Oved et al. controlled the *in vitro* reactivity of the TIL by manipulating their subpopulation composition, enabling to turn nonreactive TIL into reactive ones and vice versa, by simple depletion of specific subpopulation. Measuring the subpopulation frequencies of TIL from 45 ACT treated patients is performed these days at our melanoma research center. Frequencies of hundreds of subpopulations will be determined by flow cytometry and the correlation to clinical response will be evaluated by a computational model. The result of this study will be of great interest as it may reveal the TIL characterization profile of responding patients.

The combination of T cell transfer with monoclonal antibodies blocking CTLA-4 or PD-1 function should also be evaluated in future studies. CTLA-4 and PD-1 are known to hinder T cell function via blockade of costimulatory signals. Therefore, addition of those antibodies could avoid the inhibitory effects of CTLA-4 or PD-1 on transferred T cells.

A lot of efforts have been invested in the field of genetic engineered lymphocytes utilizing genes encoding *αβ*TCR and CAR to target TAA. Genetic-engineering also enables the production of T cells with other functions besides TAA recognition. Modified T cells might constantly express costimulatory molecules like CD28, produce autocrine cytokines such as IL-2, express homing molecules (e.g., CD62L) or prevent apoptosis by the expression of antiapoptotic genes (e.g., bcl-2). Genetic approaches provide a platform for further developments in immunotherapy and will direct ACT to a new era of antitumor therapy.

Another important aim is the reduction of the side effects related to the treatment. Establishing cells for ACT at the laboratory is challenging, but great efforts are required from clinicians as well. Most grade III and IV toxicities are directly related to the intravenously administration of high-dose bolus IL-2 (720,000 IU/kg, every 8 h) after cell transfer [16, 24]. Reducing the dose of IL-2 significantly decreases toxicity [82, 83]. Many current trials (Table 2) are therefore using low-dose subcutaneous IL-2 administration in order to relieve the patients. Subcutaneous low-dose IL-2 administration also minimizes the costs of the treatment as its administration does not require hospitalization. A combined approach of Young-TIL ACT with subcutaneous low-dose IL-2 should be evaluated in the future. This combination drastically simplifies the laboratory and clinical efforts and could allow the widespread applicability of ACT.

## 6. Conclusions

Melanoma has been widely studied as a target for immunotherapy because it has been considered more susceptible to immune attack than other tumors. Adoptive T cell transfer has not only shown promising clinical results in the last decade but also provides a platform for the future. In the coming years, the use of advanced protocols will surely enhance the anti-tumor reactivity of transferred cells. Sophisticated genetic approaches are of great potential in the future, but so far TIL ACT is the most effective cell therapy for metastatic melanoma patients, with objective responses in more than 50% of the patients. Simplifying the TIL production process, by using Young-TIL, may enable cancer centers worldwide to implement this effective approach and expose more patients to ACT therapy.

## Figures and Tables

**Table 1:** Comparison of published adoptive T cell transfer studies for melanoma patients.

	T cell type	Origin of T cell	Culture initiation	*In vitro* screening	Large-scale expansion protocol	Patient conditioning	OR (%)	Ref
A	TIL	Melanoma biopsy	One single bulk culture	None	Cells grown with IL-2 till desired number obtained	Cyclophosphamide	35	[24, 25]
B	Selected-TIL	Melanoma biopsy	Multiple cultures	IFN*γ* ELISA	anti-CD3, IL-2, IFC for 14 days	NMC	50	[17, 41]
C	Young-TIL	Melanoma biopsy	One single bulk culture	None	anti-CD3, IL-2, IFC for 14 days	NMC	50	[16]
D	Selected-TIL	Melanoma biopsy	Multiple cultures	IFN*γ* ELISA	anti-CD3, IL-2, IFC for 14 days	NMC + 2 Gy TBI	52	[18]
E	Selected-TIL	Melanoma biopsy	Multiple cultures	IFN*γ* ELISA	anti-CD3, IL-2, IFC for 14 days	NMC + 12 Gy TBI	72	[18]
F	Engineere T cells	Leukopheresis	Gene therapy with low-affinity MART-1 TCR	IFN*γ* ELISA, Transductio efficiency	various protocols	NMC	12	[56]
G	Engineere T cells	Leukopheresis	Gene therapy with high-affinity MART-1 TCR	IFN*γ* ELISA, Transductio efficiency	various protocols	NMC	30	[60]
H	Engineere T cells	Leukopheresis	Gene therapy with gp100 TCR	IFN*γ* ELISA, Transductio efficiency	various protocols	NMC	19	[60]

(TIL) Tumor infiltrating lymphocytes, (OR) objective response, (Ref) Reference, (IFC) irradiated feeder cells, (NMC) nonmyeloablative chemotherapy, and (TBI) total body irradiation.

**Table 2:** Adoptive T cell transfer trials currently recruiting patients.

NCT ID	Sponsors	Phase	Study design	Pat. No.	Transferred cells	TBI	NMC	IL-2
00287131	Sheba Medical Center	II	NR	20	Selected or Young-TIL	−	+	+
00338377	M.D. Anderson Cancer Center Chiron Corporation	II	R	83	TIL versus TIL with dendritic cell immunization	−	+	+
01118091	National Cancer Institute	II	R	135	IL-2 versus IL-2 with enriched Young TIL CD8	−	+	+
00513604	National Cancer Institute	II	NR	149	TIL +/− CD4+ depletion	−	+	+
00910650	University of California; California Institute of Technol. Univer. of Southern California University of Connecticut	II	NR	22	anti-MART-1TCR-gene engineered lymphocytes and MART-1 peptide pulsed dendritic cells	−	+	+
00612222	National Cancer Institute	II	NR	41	anti-MART-1 TCR-gene engineered lymphocytes and ALVAC virus immunization	−	+	+
00610311	National Cancer Institute	II	NR	41	anti-gp100 TCR-gene engineered lymphocytes and ALVAC virus immunization	−	+	+
00512889	Dana-Farber Cancer Institute	I	NR	20	MART1/Melan-A specific CTL +/− GM-CSF and irradiation of cutaneous tumor lesion	−	−	−
01082887	Nantes University Hospital	I/II	NR	12	TIL in combination with intra-tumoral injections of IFN*γ* adenovirus (Ad-IFN*γ*)	−	−	+ (s.c)
00324623	Centre Hospitalier Universitaire Vaudois	I	NR	12	Melan-A/MART-1 antigen-specificCD8 T lymphocytes	−	+	−
00814684	National Cancer Institute	II	R	95	anti-Mart-1 and peptide vaccines versus anti-gp100 TCR-gene engineered lymphocytes and peptide vaccines	+	+	−
00670748	National Cancer Institute	II	NR	82	anti-NY ESO-1 TCR-Gene engineered lymphocytes	−	+	−
01029873	Altor Bioscience Corporation; National Cancer Institute	I/II	NR	58	Cisplatin with ALT-801 (IL-2 fused to T-cell receptor directed against p53-derived peptides)	−	−	−
00393029	National Cancer Institute	II	NR	12	anti-p53 TCR-gene engineered lymphocytes	−	+	+
00871481	Fred Hutchinson Cancer Research Center; National Cancer Institute	I/II	∗∗∗	30	Autologous NY-ESO-1-melanoma-specific CD8^+^ T cells +/− ipilimumab	−	+	+ (s.c)
01106235	Fred Hutchinson Cancer Research Center	I	NR	10	Autologous IL-21 modulated CD8^+^ antigen-specific T cells	−	+	+ (s.c)
00925314	Cosmo Bioscience	II	NR	20	CB-10-01 (transgenic lymphocyte immunization against telomerase)	−	−	−
00062036	National Cancer Institute	I/II	NR	33	IL-2 gene-transduced TIL	−	+	+
00924001	National Cancer Institute	I/II	NR	30	Allogeneic tumor-reactive lymphocyte cell line DMF5	−	+	+

All clinical trials are registered on http://www.clinicaltrials.gov/; (NCT ID) Clinical trial identifier, (Pat. No.) Number of treated patients, (R) Randomized, (NR) none randomized, (***) not reported, (TIL) tumor infiltrating lymphocytes, (TBI) total body irradiation, (NMC) nonmyeloablative chemotherapy, (s.c) subcutaneous.
